# A panel of recombinant proteins for the serodiagnosis of caseous lymphadenitis in goats and sheep

**DOI:** 10.1111/1751-7915.13454

**Published:** 2019-07-09

**Authors:** Thiago Doria Barral, Ricardo Barros Mariutti, Raghuvir Krishnaswamy Arni, Anderson Jesus Santos, Dan Loureiro, Ana Rita Sokolonski, Vasco Azevedo, Sibele Borsuk, Roberto Meyer, Ricardo Dias Portela

**Affiliations:** ^1^ Laboratório de Imunologia e Biologia Molecular ICS‐UFBA Salvador BA 40110‐100 Brasil; ^2^ Centro Multiusuário de Inovação Biomolecular UNESP São José do Rio Preto SP 15054‐000 Brasil; ^3^ Laboratório de Genética Celular e Molecular ICB‐UFMG Belo Horizonte MG 31270‐901 Brasil; ^4^ Laboratório de Biotecnologia Infecto‐parasitária CDT‐UFPel Pelotas RS 96010‐900 Brasil

## Abstract

Caseous lymphadenitis (CLA) is a small ruminant disease characterized by the development of granulomatous lesions in superficial and internal lymph nodes, as well as in some organs, and causes significant economic losses worldwide. The aetiological agent of CLA is the bacterium *Corynebacterium pseudotuberculosis*; however, the commercially available diagnostic tools present problems with regard to specificity, which can lead to false‐negative results. This study aimed to develop an indirect enzyme‐linked immunosorbent assay (ELISA) for the detection of specific immunoglobulins in goats and sheep using recombinant *C. pseudotuberculosis* PLD, CP40, PknG, DtxR and Grx proteins. For validation of the ELISAs, 130 goat serum samples and 160 sheep serum samples were used. The best ELISA for goats was developed using a combination of PLD and CP40 as antigens at a 1:1 ratio, which presented 96.9% sensitivity and 98.4% specificity. The most effective ELISA for sheep presented 91% sensitivity and 98.7% specificity when recombinant PLD alone was used as the antigen. These ELISAs can be used as highly accurate tools in epidemiological surveys and for the serodiagnosis of *C. pseudotuberculosis* infection in goats and sheep.

## Introduction

*Corynebacterium pseudotuberculosis*, a bacterium characterized as a facultative anaerobic, fermentative irregular and Gram‐positive coccobacillus (Dorella *et al*., 2006), causes several diseases with major economic importance including caseous lymphadenitis (CLA) in goats and sheep and ulcerative lymphangitis in horses and cows (Connor *et al*., 2007). *Corynebacterium pseudotuberculosis* can also affect humans (Bastos *et al*., 2011). CLA causes significant economic loss in small ruminant farming worldwide, mainly owing to the decreased production of milk, wool and meat, along with reduction in the commercial value of leather (Dorella *et al*., 2006). Moreover, because the disease has a long incubation period of two to six weeks, the segregation of infected and not‐infected animals is difficult, with severe implications for disease control in breeding flocks (Chirino‐Zárraga *et al*., 2006).

The gold standard for CLA diagnosis is the isolation of the causative agent from abscesses; however, it is not always possible to collect these samples. First, CLA can only be clinically detected when superficial lymph nodes are affected, whereas a proportion of infected animals, mainly sheep, present lesions that only affect internal lymph nodes and organs such as the liver, spleen and kidney. Thus, infected animals can remain in the flock to serve as a source of infection (Soares *et al*., 2012). Second, bacteria from these lesions may be not viable for *in vitro* isolation and growth (Ribeiro *et al*., 2013), and the use of polymerase chain reaction (PCR) amplification for the direct detection of the bacteria in the abscess material can be hampered by the enzymes found in these lesions or even by DNA degradation, as already described by Pacheco *et al*. (2007). Although immunoassays such as enzyme‐linked immunosorbent assay (ELISA) are able to detect animals carrying the internal form of the disease (Dercksen *et al*., 2000), many of the assays developed to date are unable to provide high sensitivity and specificity values (Malone *et al*., 2006; Stapleton *et al*., 2009). Notably, assays presenting deficiencies in sensitivity can also contribute to the spread of disease owing to misdiagnosis of positive animals (Sunil *et al*., 2008), whereas the lack of specificity can lead to the euthanasia of livestock that are not actually infected with *C. pseudotuberculosis*.

Corynebacterial phospholipase D (PLD), which was identified prior to the sequencing of the bacterial genome, is the main exotoxin produced by *C. pseudotuberculosis* and its immunodominant antigen (Songer *et al*., 1988). This enzyme is able to hydrolyse sphingomyelin, leading to weakening of the host cellular membrane and favouring the infection process (Pépin *et al*., 1989). Several immunoassays have already been developed using PLD as an antigen, which proved to be well suited for this application (Sting *et al*., 2017). Nevertheless, antigens best suited for indirect ELISA should also be considered. For example, the *C. pseudotuberculosis* CP40 protein, identified by Walker *et al*. (1994) and recently described as an endo‐β‐N‐acetylglucosaminidase (Shadnezhad *et al*., 2016), is characterized as a 40 KDa secreted factor that is highly recognized by the sheep immune system in the acute phase of the disease (Silva *et al*., 2014).

Sequencing and subsequent analyses of the genomes of more than 70 strains of *C. pseudotuberculosis* have provided information regarding the molecular and genetic basis of bacterial virulence and led to the discovery of new and promising antigens (Rezende *et al*., 2016; Brum *et al*., 2017). In particular, the FRC41 strain genome contains a gene that codes for protein kinase G (PknG) (Trost *et al*., 2010), although its specific role remains to be determined (Santana‐Jorge *et al*., 2016). This molecule attracted specific interest because a homologous protein from *Mycobacterium tuberculosis* blocks fusion between the host macrophage phagosome and the lysosome during phagocytosis (Walburger *et al*., 2004; Warner and Mizrahi, 2007). Notably, it had already been revealed that PknG presents significant B‐cell epitopes (Santana‐Jorge *et al*., 2016). *Corynebacterium pseudotuberculosis* is also able to produce a low molecular weight oxidoreductase called glutaredoxin (Lillig and Berndt, 2013), which participates in several cellular functions, such as signal transduction and the defence against oxidative stress (Prinz *et al*., 1997). Trost *et al*. (2010) also described the presence in *C. pseudotuberculosis* of a gene that codes for a diptheric toxin repressor homologue, termed DtxR. The main role of this protein is to regulate iron absorption and inhibit toxin production in *Corynebacterium diphtheriae* and *Corynebacterium glutamicum* (Deng and Zhang, 2015).

Thus, the use of biotechnology tools, such as next‐generation DNA sequencing and bioinformatics analysis, can lead to the identification of new antigens that may be crucial in the development of vaccines and immunoassays (Galvão *et al*., 2017). The aim of this study was to use a panel of recombinant antigens for the development of a highly accurate immunoassay for the detection of this bacterial infection in small ruminants.

## Results

### Recombinant proteins expression, purification and antigenicity

**Figure 1:**
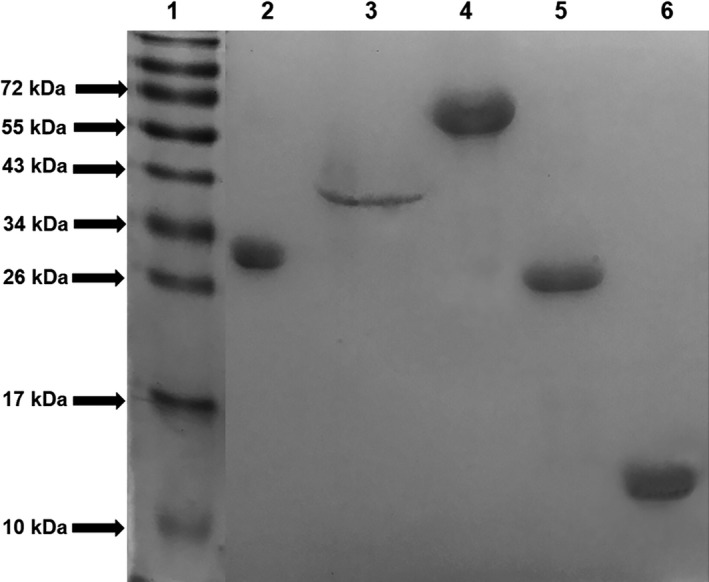
Identification of the correct expression and purification of the recombinant proteins. 50 μg of each recombinant protein was subjected to 12.5% SDS‐PAGE and stained with Coomassie blue after a 1 h run at 100V. **(1)** Molecular weight standard; **(2)** PLD;** (3)** CP40; **(4)** PknG; **(5)** DtxR; **(6)** Grx. The numbers at the side express the molecular weight values of the standard in kDa.

**Figure 2:**
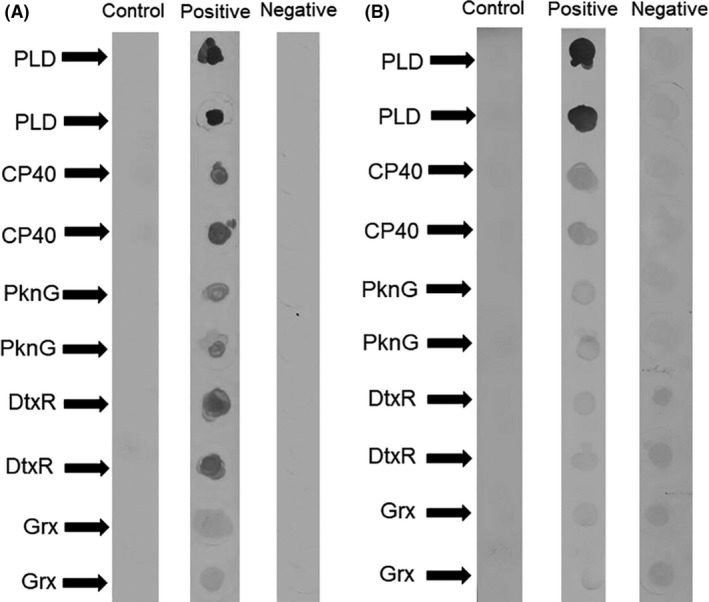
Recognition of the recombinant proteins by sera of *Corynebacterium pseudotuberculosis*‐infected and non‐infected small ruminants. The recombinant proteins PLD, CP40, PknG, DtxR and Grx were immobilized on nitrocellulose membranes in duplicate, followed by incubation with a pool of negative or positive control samples. (A) Incubation with goats’ serum sample pools and (B) incubation with a pool of positive or negative samples taken from sheep. For each recombinant protein, a blank control was included for which there was no incubation with serum.

The recombinant proteins PLD, CP40, PknG, DtxR and Grx were successfully expressed and purified, presenting molecular weights of approximately 30, 40, 69, 25 and 13 kDa in sodium dodecyl sulfate–polyacrylamide gel electrophoresis (SDS‐PAGE) respectively (Fig. 1). The antigenicity of the recombinant proteins was evaluated by a dot‐blot assay, using positive and negative serum sample pools. All the proteins were recognized by goats that were infected by *C. pseudotuberculosis* (Fig. 2A), with an intense reaction produced by PLD, CP40 and DtxR, along with weak recognition of these proteins by the negative pool (non‐infected control goats). When the same protocol was employed to verify recognition of the proteins by sheep antibodies, PLD exhibited a greater reaction with antibodies from CLA‐infected sheep, albeit no reaction with the negative serum sample pool. It was also observed that the sheep negative serum sample pool significantly reacted with the DtxR and Grx proteins (Fig. 2B).

### PLD or PLD plus CP40 promotes the most effective discrimination between the optical density results of the positive and negative serum sample pools

For the ELISA development and standardization, we performed a checkerboard system assay, wherein different positive and negative serum samples, antigen and anti‐antibody conjugate dilutions were tested in combination. For goats, when PLD alone (ratio = 51.14) and the combination with CP40 (ratio = 76.08) were used as antigens, the discrimination between the optical densities (ODs) of the positive and negative serum samples was greater (Table**** 1). The ratios calculated for the assays using sheep serum pools were lower, whereas the use of PLD (ratio = 22.34), PLD and CP40 (ratio = 17.62), and PknG (ratio = 13.76) as antigens resulted in the highest ratios, as seen in Table**** 1. The control positive and negative serum samples were then tested in ELISAs using the recombinant proteins that provided a ratio between the positive and the negative serum sample pool OD results > 5.0.

**Table 1:** Ratio between positive and negative serum sample pool OD values obtained in indirect ELISAs using different *C. pseudotuberculosis* recombinant antigens. The antigens used were the recombinant proteins PLD, CP40, a combination 1:1 of PLD, and CP40, PknG, DtxR and Grx for each species. The optimal conditions were obtained through a checkerboard titration methodology, in which different antigen concentrations, serum sample pools and conjugated antibody dilutions were tested

Species	Recombinant protein	Pos/Neg OD ratio	Serum dilution	Anti‐IgG dilution	Protein concentration
Goats	PLD	51.14	1:200	1:10 000	2 μg ml^−1^
	CP40	5.94	1:200		5 μg ml^−1^
	PLD + CP40	76.08	1:400		2 μg ml^−1^ each
	PknG	1.71	1:50		0.25 μg ml^−1^
	DtxR	3.67	1:200		2 μg ml^−1^
	Grx	3.15	1:50		0.25 μg ml^−1^
Sheep	PLD	22.34	1:400	1:5000	2.5 μg ml^−1^
	CP40	9.02	1:50		2.5 μg ml^−1^
	PLD + CP40	17.62	1:400		5 μg ml^−1^ each
	PknG	13.76	1:50		1 μg ml^−1^
	DtxR	3.08	1:50	1:10 000	2.5 μg ml^−1^
	Grx	2.91	1:50		2.5 μg ml^−1^

### PLD or PLD plus CP40 provides high sensitivity and specificity for the serodiagnosis of CLA

**Figure 3:**
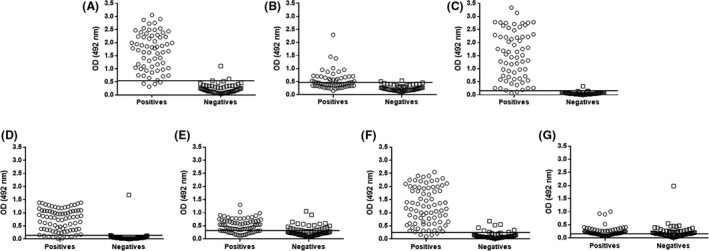
Individual distribution of the OD results of the negative and positive control serum samples. For positive and negative control samples taken from goats, we used the recombinant proteins (A) PLD, (B) CP40 and (C) PLD and CP40 in a 1:1 combination. For negative and positive control samples from sheep, we used the recombinant proteins (D) PLD, (E) CP40, (F) PLD and CP40 in a 1:1 combination, and (G) PknG. The graphs indicate the OD values obtained for each positive or negative sample using each recombinant protein. The lines within the graphs represent the cut‐off value for each assay.

When control serum samples were individually tested in each indirect ELISA (iELISA) (Fig. 3)**,** it was observed that for goats, two false‐positive results (negative samples above the cut‐off) occurred in the assay using PLD or CP40 as antigens. In the assay using a 1:1 combination of these two proteins, only one false‐positive result was noted. Regarding the false‐negative results (positive control sera presenting OD values below the cut‐off), it was observed that the assay using only PLD resulted in four false negatives and that the PLD:CP40 assay presented only two false negatives. The assay using only CP40 as the antigen produced several false‐negative results. For sheep, seven false negatives were detected using only PLD as the antigen, whereas the combination PLD:CP40 produced six such results. The low reactivity of sheep serum samples to PknG and CP40 alone resulted in a considerable number of false‐negative results.

**Figure 4:**
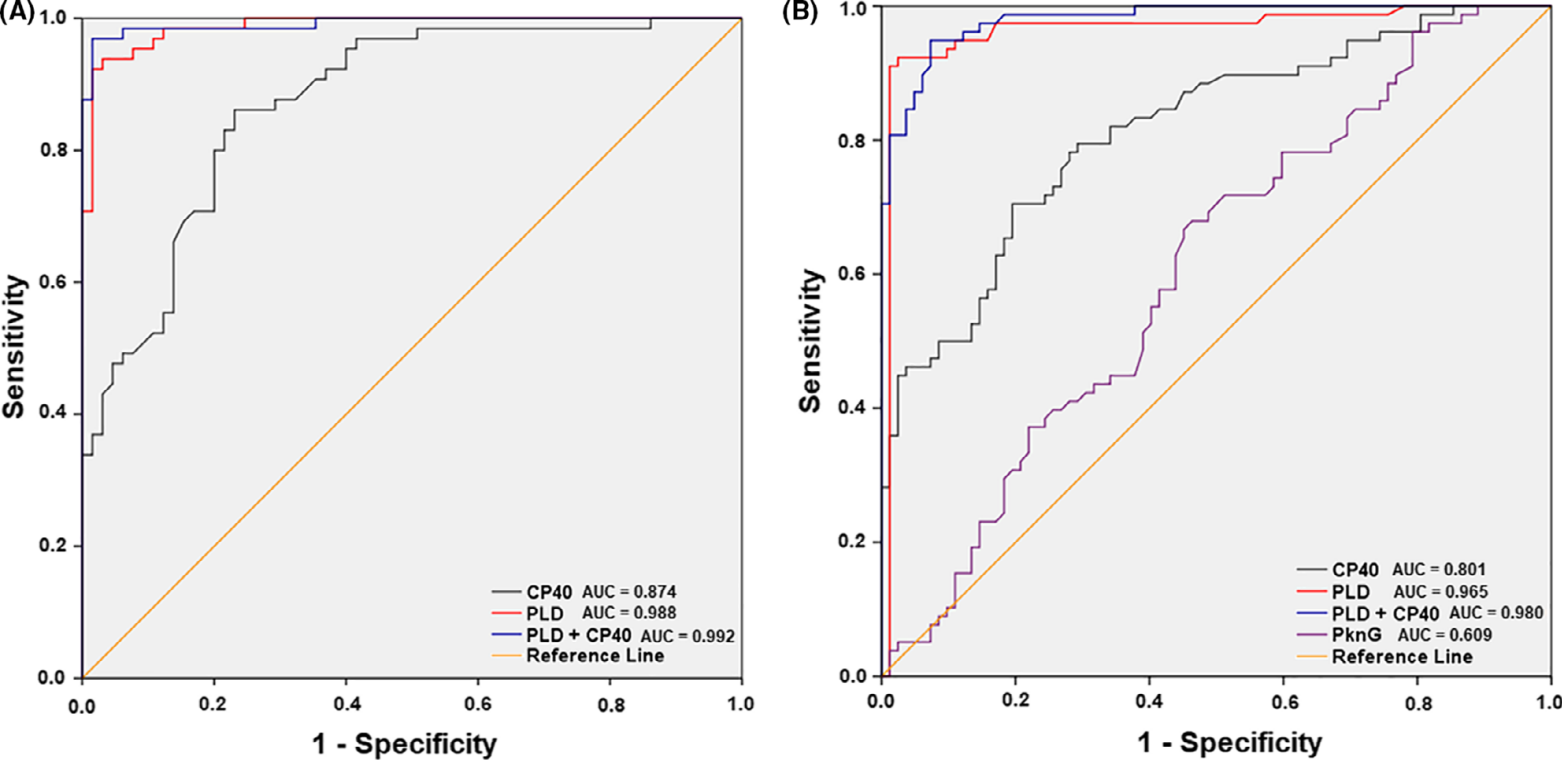
Receiver operating characteristic (ROC) curves for the developed indirect ELISAs. For generation of the ROC curves, we used (A) negative and positive control serum samples that were taken from goats and tested based on an ELISA using the recombinant proteins PLD, CP40 and a combination 1:1 of PLD and CP40; and (B) negative and positive control serum samples that were taken from sheep and tested using an ELISA with the recombinant proteins PLD, CP40, a combination 1:1 of PLD and CP40, and PknG as antigens.

The results of the validation study of these ELISAs, developed for detecting infection by *C. pseudotuberculosis* in goats, are shown in Table**** 2. The assay using the PLD:CP40 combination as antigens showed the best combined sensitivity and specificity, of 96.9% and 98.4%, respectively, with 99.2% accuracy, as shown by the area under the curve (AUC) of the receiver operating characteristic (ROC) curve (Fig. 4A). The assay also provided high reproducibility and repeatability and a *Kappa* index of 0.954. Regarding the parameters attained for the assays developed for sheep (Table**** 3), the assay using PLD alone as the antigen produced 98.7% specificity, 91% sensitivity and a *Kappa* index of 0.900, with 96.5% accuracy, as shown by the AUC of the ROC curve (Fig. 4B). When the PLD:CP40 combination was used for testing the sheep serum samples, 94.8% specificity and 92.6% sensitivity were observed, with 98% accuracy and a *Kappa* index of 0.875. The assay using PknG as the antigen produced validation parameters with lower values than those that had been previously described in this study.

**Table 2:** Validation parameters for goat indirect ELISAs using different recombinant antigens. *C. pseudotuberculosis* recombinant proteins PLD, CP40, and a combination 1:1 of the two proteins were used as antigens. The conditions for the ELISA procedures were those that presented the best discrimination between the positive and negative serum sample pool optical densities (ODs)

Parameter	PLD	CP40	PLD + CP40
Positive control	65	65	65
Negative control	65	65	65
False positives	2	2	1
False negatives	4	37	2
Cut‐off (Optical density)	0.554	0.464	0.159
Sensitivity (%)	93.8	43	96.9
Specificity (%)	96.9	96.9	98.4
Accuracy (%)	98.8	87.4	99.2
Positive predictive value (%)	96.8	93	98.4
Negative predictive value (%)	94	63	96.9
Repeatability positive control (%)	98	99.6	97.3
Repeatability negative control (%)	99.8	99.9	99.9
Repeatability serum with OD result close to cut‐off (%)	99.8	99.9	99.8
Reproducibility (%)	100	100	100
*Kappa*	0.908	0.400	0.954

**Table 3:** Validation parameters for the sheep indirect ELISAs. *C. pseudotuberculosis* recombinant proteins PLD, CP40, a combination 1:1 of these two proteins, and PknG were used as antigens. The conditions for the ELISA procedures were those that presented the best discrimination between the positive and negative serum sample pool optical densities (ODs)

Parameter	PLD	CP40	PLD + CP40	PknG
Positive control	78	78	78	78
Negative control	82	82	82	82
False positives	1	22	6	38
False negatives	7	19	4	26
Cut‐off (Optical density)	0.144	0.306	0.261	0.166
Sensitivity (%)	91	75.6	94.8	66.7
Specificity (%)	98.7	73	92.6	53.6
Accuracy (%)	96.5	80.1	98	60.9
Positive predictive value (%)	98.6	72.8	92.5	57.7
Negative predictive value (%)	92	75.9	95	62.8
Repeatability positive control (%)	99.9	99.8	99.8	99.9
Repeatability negative control (%)	99.9	99.9	99.9	99.9
Repeatability serum with OD result close to cut‐off (%)	99.9	99.9	99.9	99.9
Reproducibility (%)	100	100	100	100
*Kappa*	0.900	0.488	0.875	0.215

### Western blot confirms that PLD and CP40 are specifically recognized by animals infected by *Corynebacterium pseudotuberculosis*

**Figure 5:**
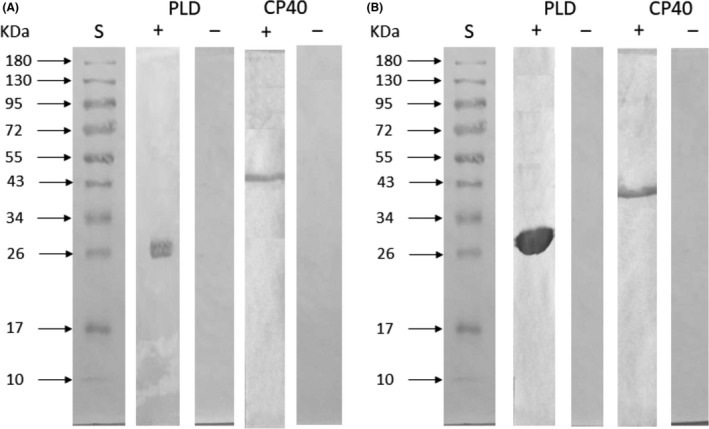
Western blot assay for analysis of the recognition of recombinant proteins. The recombinant proteins were resolved by 12.5% SDS‐PAGE, transferred to a nitrocellulose membrane, and then incubated with a pool of positive and negative control serum samples from (A) sheep and (B) goats. (S) Molecular weight standard. The numbers at the side express the molecular weight values of the standard in kDa.

The Western blot analysis (Fig. 5) confirmed that both PLD and CP40 are significantly recognized by the serum samples from *C. pseudotuberculosis*‐infected goats and sheep and that there is no other molecule in the protein solution that reacts with the antibodies presented in the samples. In addition, the negative serum sample pool did not react with either of these two recombinant proteins.

### iELISA application as an epidemiological test

The assays developed in this study were compared to those developed by Rebouças *et al*. (2013) and Seyffert *et al*. (2010) using serum samples taken in an epidemiological survey of sheep and goats from Sergipe, a Brazilian State. The goat assay using PLD:CP40 was able to identify 38 positive samples (55.8%), whereas the assay using total secreted/excreted antigen detected 30 positive samples (44.11%). For sheep, the assay using the recombinant PLD detected 236 positive samples (50%), whereas the assay using secreted/excreted antigen produced 208 positive results (44.06%). Also, from nine sheep with caseous lesions, eight were positive for the secreted/excreted antigen ELISA, and all of them were positive at the ELISA using PLD as antigen. All of the five goats with caseous lesions were positive in the secreted/excreted antigen ELISA and in the ELISA using PLD and CP40 as antigens.

## Discussion

Caseous lymphadenitis constitutes a disease of considerable economic relevance for small ruminant breeders worldwide. Currently available diagnostic tools provide limited efficacy. Recent analysis of the *C. pseudotuberculosis* pangenome allowed the discovery of new bacterial proteins presenting B‐cell epitopes (Santana‐Jorge *et al*., 2016), and another study provided new insights in the use of the CP40 protein as an immunogenic through bioinformatics analysis (Droppa‐Almeida *et al*., 2018). By employing some of these newly discovered antigens in the development of immunoenzymatic assays, the present work demonstrated that several of these molecules are broadly recognized by the host immune system and thus can be used to support accurate immunodiagnostic assays.

Although antibodies from *C. pseudotuberculosis*‐infected animals recognized all five of the tested proteins, standardization of the immunoenzymatic assays revealed that Grx and DtxR did not possess high sensitivity or specificity and were unable to satisfactorily discriminate between samples from infected and non‐infected animals. These findings confirmed the results of the dot‐blot assay, in which these proteins produced a less intense recognition pattern associated with reactivity to a pool of negative serum samples, whereas PLD and CP40 showed an intense reaction by Western blot, without reaction with the negative serum sample pool. This is consistent with previous findings that the proteins of the Grx family are present in a wide variety of microorganisms and are essential to many biochemical pathways (Eberle *et al*., 2018). Accordingly, antibodies against these proteins could be generated when animals become infected by those microorganisms, creating serological cross reactivity. In contrast, the gene responsible for expression of the DtxR protein has been found mainly in *C. pseudotuberculosis* strains isolated from buffalo (Selim *et al*., 2016). This gene may, therefore, be absent from strains that affect the majority of small ruminant herds and therefore may lead to false‐negative serological results. Moreover, the assay using the PknG protein did not produce satisfactory results, due to low sensitivity and specificity. Conversely, Santana‐Jorge *et al*. (2016) previously described that this protein has a significant number of B‐cell epitopes. The present *in vivo* result, which differed from the predictions of the bioinformatics analysis, may result from low expression of the antigen in clinical isolates of the bacteria, or because most bioinformatics software packages base their analyses on murine and human immunological data. To date, no bioinformatics tool specific for small ruminants is available.

Notably, we obtained significant results with regard to sensitivity and specificity for some of the developed assays. The use of CP40 as an antigen for the serodiagnosis of caseous lymphadenitis has not previously been described before, although this protein has been used in vaccine trials, offering partial protection against *C. pseudotuberculosis* virulent challenge (Silva *et al*., 2014; Droppa‐Almeida *et al*., 2016). In particular, Menzies *et al*. (2004) achieved 97% sensitivity and 81% specificity when using a recombinant PLD. The present study produced higher sensitivity and specificity values due to the best mode used to standardize the ELISAs developed herein. Furthermore, CP40 in combination with PLD in a 1:1 ratio yielded the highest sensitivity and specificity values for testing of goat sera. In comparison, Seyffert *et al*. (2010) employed native secreted/excreted antigens and reached 98.5% specificity and 93.5% sensitivity. Our improved sensitivity results thus point to an enhanced concentration of immunodominant molecules in the recombinant protein solution and therefore being most effective to achieve positive results in serum samples from low responder animals.

Hoelzle *et al*. (2013) described that 100% of the 60 goats tested by Western blot recognized the native PLD protein, whereas in the present study, 4 of the 65 animals failed to recognize the recombinant PLD. These differences in the recognition of the protein may be a consequence of the strain used for antigen production, which might produce a PLD with different amino acid sequences, or even to the different serodiagnosis assay employed. Moreover, Sting *et al*. (2017) described the use of iELISA for goats, incorporating whole cell antigens or recombinant PLD, with results reaching a maximum of 91% sensitivity and 100% specificity. In comparison, our ELISA using PLD and CP40 presented a lower specificity (98.4%), albeit a higher sensitivity (96.9%).

The use of PLD protein alone generated the most promising immunoassay for sheep, reaching 98.7% specificity and 91% sensitivity, with 96.5% accuracy. The assay using the PLD:CP40 combination yielded validation parameter values very close to those of the assay using PLD protein alone, the best ratio of positive/negative control ODs, a low cut‐off value and lower OD values for negative control samples, demonstrating that the PLD assay was able to better discriminate between positive and negative results. The sheep assay using PLD only was also more effective than the immunoenzymatic assay developed by Rebouças *et al*. (2013), which used crude *C. pseudotuberculosis* secreted/excreted molecules as antigens, achieving 99% specificity and 89% sensitivity. Notably, Solanet *et al*. (2011) achieved 100% specificity and 98% sensitivity using bacterial somatic antigens in an immunoenzymatic platform; however, this work was conducted using samples from 17 animals that underwent an experimental infection, and the bacterial strains used for obtaining the antigen were the same as those used for the challenge. Hoelzle *et al*. (2013) also performed a Western blot assay with the serum samples from 40 animals, observing that 71% recognized the PLD antigen, whereas in our work 71 among 78 animals (91.02%) recognized the recombinant PLD. It is likely that this difference also arose from the different immunoassays employed between the studies, as ELISA uses a more concentrated solution of the specific protein than Western blot.

The assays for goats and sheep that presented the best performances were subsequently used for an epidemiological survey of *C. pseudotuberculosis* infections in the state of Sergipe, Brazil. It was found that the assays developed using recombinant proteins were able to detect more positive samples than those using *C. pseudotuberculosis* crude secreted/excreted antigens, as described by Seyffert *et al*. (2010) and Rebouças *et al*. (2013) for goats and sheep, respectively, which are characterized by 93.5% sensitivity and 98.5% specificity (Seyffert *et al*., 2010), and 89% sensitivity and 99% specificity (Rebouças *et al*., 2013). This result confirmed the highest relative sensitivity of the assays developed in the present study. Recombinant proteins are normally employed with the objective of enhancing immunodiagnostic assays, specificity owing to their purified state and the lower presence of proteins that can cross‐react with other molecules from infectious microorganisms. Recombinant proteins can also influence sensitivity, as the amount of immunodominant proteins that are used to sensitize plates is higher than the concentration of these specific molecules in a crude antigen extract.

In conclusion, the iELISAs developed in the present study using recombinant CP40 and PLD were able to discriminate between positive and negative serum samples with high sensitivity and specificity. They therefore represent promising tools that can be used in epidemiological surveys and caseous lymphadenitis control programs, owing to their high accuracy in the detection of goats and sheep infected by *C. pseudotuberculosis*.

## Experimental procedures

### Animal serum sample acquisition

With the objective of establishing the optimal experimental conditions and to determine the validation parameters of the assays developed for each protein, we used serum samples from 290 animals. Accordingly, positive control samples were taken from 65 goats and 78 sheep for which *C. pseudotuberculosis* infection was confirmed by isolation of the bacteria from caseous lesions. As negative controls, we used samples from 65 goats and 82 sheep sourced from caseous lymphadenitis non‐endemic areas in Southern Brazil, where there is a strict control in the introduction of new animals. The epidemiological survey was performed through the collection of samples from small ruminants from Sergipe State, Brazil, with samples taken from 63 goats and 472 sheep being included in this study. The serum sample collection for this study was approved by the Committee on Ethics in the Use of Animals in Experiments of the School of Veterinary Medicine of the Federal University of Bahia (protocols numbers 35/2017 and 89/2017).

### Cloning, expression and purification of recombinant proteins

The sequences encoding the proteins DtxR, Grx, PLD and PknG were synthesized by GenScript (Piscataway, NJ, USA) based on the sequences deposited at the UniProt database and cloned into the vector pET28a. The UniProt IDs of the sequences are as follows: DtxR: D9QAW1; Grx: D9Q987; PLD: P20626; PknG: D9QCK3 (www.uniprot.org). The signal peptide sequences were deleted and a polyhistidine tag was added at the N‐ termini. DtxR and Grx proteins were then expressed in the *Escherichia coli* strain BL21 (DE3) T1, and PLD and PknG were expressed in the *E. coli* strain BL21 (DE3) pLysS. When transformed cell cultures reached OD values of 0.5–0.7, they were induced with 0.3 mM isopropyl β‐D‐1‐thiogalactopyranoside (IPTG; Sigma‐Aldrich, Saint Louis, MO, USA) for 16 h at 20 °C in LB medium supplemented with kanamycin (Sigma‐Aldrich). The cells were harvested by centrifugation at 8,000 × *g* for 10 min at 4 °C, resuspended in 20 mM Tris‐HCl buffer pH 8.0 containing 300 mM NaCl and lysed on ice with ten sonication pulses of 15 s each, using an ultrasonic processor (Thermo Scientific, Waltham, MA, USA), and finally centrifuged at 15 000 × *g* for 15 min. The supernatant was subjected to affinity chromatography using an immobilized nickel column (GE, Chicago, IL, USA) under native conditions. After concentration of the target protein through vacuum centrifugation, a second step of gel filtration was performed using a Superdex G 75 10/300 GL column (Sigma‐Aldrich). The protein content of recombinant protein solution was quantified using a commercial kit based on the bicinchoninic acid method (Thermo Fisher Scientific), and 50 μg of each protein was subjected to 12.5% SDS‐PAGE to determine the correct purification of the expressed proteins. Expression of the target proteins was confirmed by Western blot using an anti‐polyhistidine antibody (R&D Systems, Minneapolis, MN, USA).

The coding sequence of the *CP40* gene was amplified using the primers F5′CGC GGA TCC ATG CAT AAT TCT CCT CGA TCA G3′ and R5′ CGG GAA TTC TTA TCT AGA ACC AGT TGG CTT TC3′. For PCR, 50 ng of genomic DNA from *C. pseudotuberculosis* strain 1002 (a reference strain used for genome sequencing) and 10 μM of each primer were mixed with PCR Master Mix (Promega, Madison, WI, USA) in a total volume of 50 μl. The amplified region was cloned into the BamHI and EcoRI sites of the pAE vector using standard techniques. The recombinant clones were characterized by enzymatic digestion with BamHI and EcoRI enzymes (Promega). The pAE/cp40 recombinant plasmid was introduced by heat shock into the expression strain *E. coli* BL21 (DE3) Star. Expression was induced by the addition of 1 mM IPTG to the culture medium, followed by incubation in an orbital shaker (200 rpm) at 37 °C for 3 h. Western blotting using a monoclonal anti‐6 × histag antibody conjugated to peroxidase (Sigma‐Aldrich) was performed to confirm expression of the recombinant CP40 protein. Purification was performed by affinity chromatography using a HisTrap^™^ sepharose–nickel column (GE). Purity was determined by 12.5% SDS‐PAGE, and the protein concentration was determined as described above.

### Dot blotting

Nitrocellulose membranes (Thermo Fisher Scientific) were sensitized with 5 μl of a solution containing 10 μg of each recombinant protein, in duplicate. After the membranes had dried completely, they were transferred to centrifuge tubes and incubated overnight with a solution of phosphate buffer saline (PBS) plus 10% casein (Sigma‐Aldrich), at 4 °C under agitation. The membranes were then washed three times with PBS 0.5% Tween 20 (PBST) and incubated for 2 h under agitation with pools of positive or negative serum samples diluted 1: 2,000 in PBST 5% casein. Blank controls were incubated with PBST only. After five washes with PBST, the membranes were incubated with anti‐goat or anti‐sheep immunoglobulin conjugated with horseradish peroxidase (Sigma‐Aldrich) diluted 1: 20 000 for 2 h under agitation. After five more washes, the reaction was developed with a solution containing diaminobenzidine (Sigma‐Aldrich) and H_2_O_2_ and stopped after five min with deionized water.

### Indirect ELISAs

The ELISAs were developed based on a checkerboard procedure, as described by Rebouças *et al*. (2013), upon which we established the optimal antigen concentrations, serum samples, and conjugated antibodies dilutions. The parameters that we based on the choice of these conditions revealed optimal positive control OD/negative control OD ratios, with lower values for the blank controls. The positive and negative serum sample pools were tested at the dilutions 1:50, 1:100, 1:200 and 1: 400 and contained protein concentrations of 0.25, 0.5, 1.0, 2.0, 2.5 and 5.0 μg ml^−1^. The conjugated antibody was tested at the dilutions 1:5000, 1:10 000, 1:20 000 and 1:40 000. The same negative and positive serum sample pools were used in all the assays, to ensure their reproducibility and to normalize the OD values.

Briefly, wells of flat bottom high‐binding polystyrene microtiter plates (Costar Corning, New York, NY, USA) were sensitized with 100 μl of the recombinant proteins diluted in carbonate–bicarbonate buffer pH 9.6 for 16 h at 4 °C. After two washes with PBST, the plates were blocked with PBS 5% casein, 300 μl per well for 3 h at 37 °C. The plates were washed twice with PBST, and the serum samples were diluted in PBS 1% casein, of which 100 μl was added to each well and incubated for 1 h at 37 °C. After five washes with PBST, 100 μl of anti‐sheep or anti‐goat antibody conjugated with horseradish peroxidase (Sigma‐Aldrich) diluted 1:5,000 or 1:10 000 in PBS 1% casein, respectively, was added to the wells, followed by an incubation for 1 h at 37 °C. Finally, after five more washes with PBST, the reaction was developed using a solution of tetramethylbenzidine (Thermo Fisher Scientific) and H_2_O_2_ for 10 min, then stopped with a 2 N H_2_SO_4_ solution. The OD values were obtained photometrically (Bio‐Rad, Hercules, CA, USA) at 492 nm.

The positive and negative serum sample pools herein used were generated using the control serum samples (positive samples from animals with a confirmed *C. pseudotuberculosis* infection, and negative serum samples from animals bred in a caseous lymphadenitis non‐endemic area). After reaching the best antigen concentration, serum and secondary antibody dilutions and all positive and negative serum samples were individually tested. The assay parameters were defined based on the results of these control samples. All assays were performed using the same positive and negative controls. Serum samples were considered positive when the reaction showed an optical density (OD) higher than the mean plus two standard deviations of the OD obtained for negative controls (Patarroyo *et al*., 2002).

### Western blot

A Western blot assay was performed to verify the recognition of the expressed proteins using sera of *C. pseudotuberculosis*‐infected animals. Briefly, 50 μg of the recombinant proteins was subjected to 12.5% SDS‐PAGE and then transferred to nitrocellulose membranes. After a blocking step with 10% casein in PBS pH 7.4 for 16 h at 4 °C, the membranes were washed three times with PBST and incubated with the positive and negative control serum sample pools (diluted 1:100 in PBST with 1% casein) for 1 h under agitation at 37 °C. After three more washes with PBST, the membranes were incubated with horseradish peroxidase‐conjugated anti‐sheep or anti‐goat IgG antibodies (Sigma‐Aldrich) under agitation for 1 h. After three more washes with PBST, the membranes were incubated with an enzyme substrate and 4‐chloro‐1‐naphthol and hydrogen peroxide chromogen (Sigma‐Aldrich) for 10 min, and the reaction was stopped with ultrapure water.

### Statistical analysis

The data were analysed using SPSS software (IBM Corporation, Armonk, NY, USA), in which the ROC curves for each analysis were created. The accuracy of each test was determined on the basis of AUC. The cut‐off values were generated using the point of maximum of combined specificity and sensitivity for each ROC curve (Ren *et al*., 2018). The validation parameters were calculated as described by de Oliveira *et al*. (2011). Repeatability was calculated for the negative and positive control serum sample pools tested 20 times at the same moment and by the same operator, using the formula RP = (1 – CV) × 100, where RP stands for repeatability and CV for coefficient of variation. The reproducibility of each assay was obtained by testing the same positive and negative serum sample pools by three different operators in different moments using the same described protocol, and the final value was expressed as the percentage of concordant results. The agreement between ELISA results and the *C. pseudotuberculosis* infectivity status was calculated using the Kappa (K) index with the following classification: 0 – no concordance; 0 to 0.19 – very low concordance; 0.20 to 0.40 – weak concordance; 0.40 to 0.59 – moderate concordance; 0.60 to 0.79 – substantial concordance; 0.80–1.00 – high concordance (Landis and Koch, 1977).

## Conflict of interest

None declared.
